# Fabrication of atomic junctions with experimental parameters optimized using ground-state searches of Ising spin computing

**DOI:** 10.1038/s41598-019-52438-5

**Published:** 2019-11-07

**Authors:** Shotaro Sakai, Yosuke Hirata, Mitsuki Ito, Jun-ichi Shirakashi

**Affiliations:** Department of Electrical and Electronic Engineering, Tokyo University of Agriculture & Technology, Koganei, Tokyo 184-8588 Japan

**Keywords:** Nanoscale devices, Computational nanotechnology, Quantum physics

## Abstract

Feedback-controlled electromigration (FCE) is employed to control metal nanowires with quantized conductance and create nanogaps and atomic junctions. In the FCE method, the experimental parameters are commonly selected based on experience. However, optimization of the parameters by way of tuning is intractable because of the impossibility of attempting all different combinations systematically. Therefore, we propose the use of the Ising spin model to optimize the FCE parameters, because this approach can search for a global optimum in a multidimensional solution space within a short calculation time. The FCE parameters were determined by using the energy convergence properties of the Ising spin model. We tested these parameters in actual FCE experiments, and we demonstrated that the Ising spin model could improve the controllability of the quantized conductance in atomic junctions. This result implies that the proposed method is an effective tool for the optimization of the FCE process in which an intelligent machine can conduct the research instead of humans.

## Introduction

Electromigration (EM) has been known to degrade the structures and interconnects of integrated circuits. In recent years, however, EM has become a topic of intense interest because of its potential for fabricating nanoscale structures. Most of this work involved the investigation of EM on the nanoscale with the objective of utilizing it for the fabrication of nanogaps for single-electron transistors^1,2^, single-molecule transistors^3,4^, and atomic junctions for nanoscale switches^5–7^. EM is strongly related to the current density and temperature of the metallic conductor^8^. Typical electromigrated nanogaps formed with a single voltage ramp tend to exhibit extremely high tunnel resistances and then catastrophically break^9^. In addition, high local temperatures can be reached during EM^10^, which can cause large gap openings and residual metallic nanoparticles^11^. Recently, feedback-controlled electromigration (FCE) methods have been developed to enable temperature control^12^. The FCE process actively tunes the applied voltage in response to the changing resistance/conductance of the metal nanowires. The FCE scheme has been successfully employed to create nanogaps safely and reliably by controlling the quantum states of conductance in atomic junctions^13–15^. However, the parameters that are used in actual experimental procedures are often selected from published reports or adjusted subsequently by a trial-and-error approach, which is not only time-consuming but also highly dependent on experience. Furthermore, the scheme rarely fully utilizes the correlations between parameters because of the substantial amount of data.

Recently, the Ising spin model, which is a statistical mechanics model of magnetism invented by Wilhelm Lenz in 1920^16^, has garnered considerable interest as a potential new computing architecture. The Ising spin model can be applied to many combinatorial optimization problems^17^, and it can determine the optimal solution from large numbers of candidate solutions within short calculation times^18^. The combinatorial optimization problems are mapped to find the ground state of the corresponding Ising spin model. In recent years, hardware implementations analogous to the Ising spin model have also been proposed^18–22^. They have received considerable interest as a potential new computing paradigm for addressing a wide range of social issues. In fact, many research studies have already been reported^23–30^. Here, we show that Ising spin computing can be used for FCE parameter optimization by directly mapping the parameter selection, which is a “combinatorial optimization problem,” onto the Ising Hamiltonian. In other words, we use Ising spin computing for parameter optimization based on machine learning beyond the baseline model that finds the ground state. Our work differs from that presented in previous reports in that we apply the optimization results obtained with the Ising spin computing to actual experiments in the research domain. The ability to optimize FCE parameters using Ising spin computing is equivalent to being able to address this problem with quantum annealing machines/quantum annealers based on the Ising spin model. Compared with the method of simulated annealing^31^, the performance of quantum annealers is superior and advantageous for solving problems with energy landscapes that have complicated tunneling barriers^32^. Therefore, in the future, complex optimization problems concerned with physical phenomena are also foreseen to be solved efficiently using quantum annealers. Our approach opens new avenues for bridging the gap between key problems of parameter optimization in fabricating nanoscale devices and their implementation to Ising spin computing.

**Figure 1:**
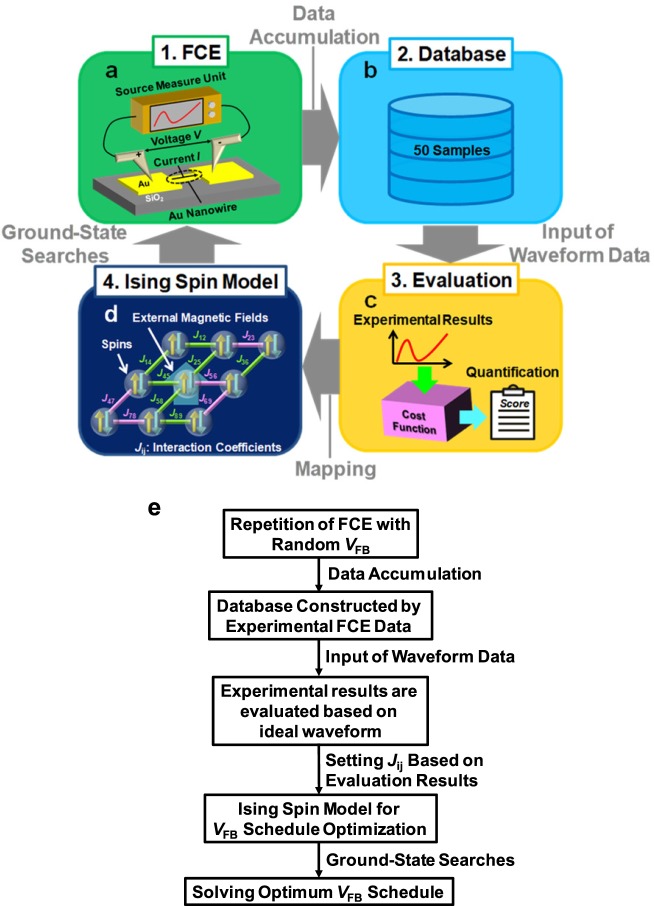
System flow for optimization of *V*_FB_ schedule. (**a**) Process of FCE with random *V*_FB_ values, with subsequent storage of the experimental data in a database. (**b**) Evaluation of experimental data by the defined cost function. (**c**) Mapping of the *V*_FB_ schedule onto the Ising spin model. (**d**) Optimization of *V*_FB_ schedule via ground-state searches of the Ising spin model, and application of the schedule to the FCE experiments. (**e**) Block diagram of system flow.

Figure 1 schematically illustrates the proposed system. We first outline our approach for constructing a database of the FCE (Fig. 1a) and then show how it is “informed” from our database (Fig. 1b). In this study, we focused on the feedback (FB) voltage *V*_FB_, which is one of the FB parameters, and collected the data of *V*_FB_. *V*_FB_ determines the amount of voltage reduction, which suppresses the heat of the channel. Therefore, this parameter plays an important role for the control of quantized conductance. In the mapping step (Fig. 1c), the problem of optimizing *V*_FB_ is mapped into the Ising spin model. The mapping step consists of two parts. One represents the solution of the original problem by the combination of binary spins and the other determines the values of the interaction coefficients and external magnetic coefficients such that the spin values at the ground state correspond to the solution of the original problem. Finally, we applied an optimum *V*_FB_ schedule obtained by the ground-state searches of the Ising spin model to the FCE experiments to confirm the usefulness of our system (Fig. 1d). In summary, Fig. 1e outlines the flow of the experiment.

## Results and Discussion

### Accumulation of FCE experimental data into database

Conventional electron-beam lithography and lift-off processes were used to fabricate Au nanowires with a constricted channel structure. First, Au nanowires were patterned on resist-coated SiO_2_/Si substrates using electron-beam lithography. Electron-beam evaporation of Ti (5 nm) and Au (20 nm) was then carried out using a developed resist pattern as a template. Finally, the unnecessary metal and resist layers were removed.

The detailed experimental steps of the FCE procedures are as follows. First, the applied voltage *V* was automatically increased with controlled loop rates in voltage steps *V*_STEP_ (=1.5 mV) while the current *I* was monitored until EM occurred. Next, when the differential conductance of the Au nanowires reached the threshold differential conductance *G*_TH_ (=20 mS), *V*_FB_ automatically reduced the applied voltage *V*, in which the voltage was reduced by 10–90% of the existing applied voltage value to slow down the EM-induced breaking process. Note that the differential conductance is obtained between measurement points and compared with *G*_TH_ during the FCE process. Finally, the applied voltage *V* was ramped up again for the next EM cycle. In this procedure, we designed the FCE schedules with randomly varying values of *V*_FB_. These schedules were applied to 50 Au nanowires, and the FCE data were stored in a database.

### Evaluation of FCE experimental parameters using cost function

**Figure 2:**
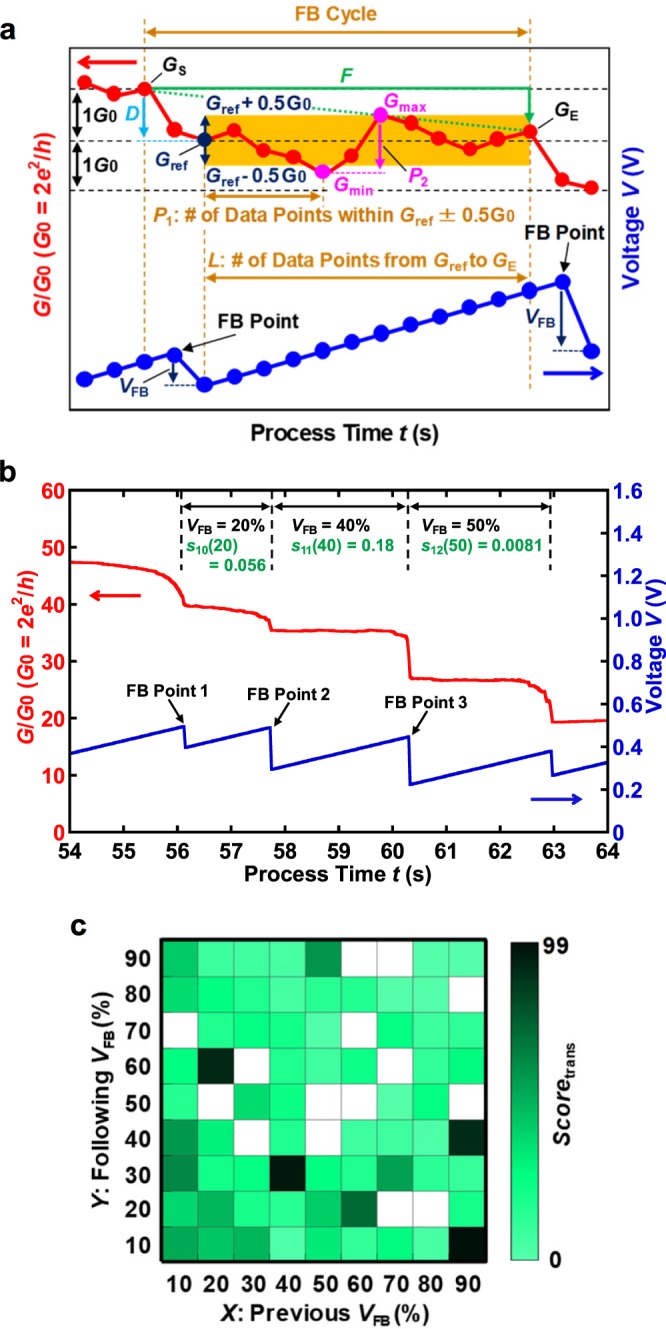
Construction of database and evaluation of *V*_FB_. (**a**) Schematic of conductance trace and each variable for cost function, where *D* = *G*_S_ − *G*_ref_, *P*_1_ is the number of data points that meet the condition of |*G* − *G*_ref_| < 0.5 *G*_0_, *P*_2_ = *G*_max_ − *G*_min_, *F* = *G*_S_ − *G*_E_, and *L* is the number of data points from *G*_ref_ to *G*_E_. (**b**) Typical time evolutions of the junction conductance, *G*/*G*_0_ (red), where *G*_0_ = 2*e*^2^/*h*, and th*e* junction voltage, *V* (blue), with random *V*_FB_ values. The evaluation is from the viewpoint of controllability of the quantized conductance using Eq. (1) at three FB points between 54 and 64 s. A larger *Score*_*V*FB_, which is infinite in an ideal case, indicates a higher evaluation. FB point 2 indicates that the quantized conductance smoothly plateaus, and Eq. (1) gives the high *Score*_*V*FB_. (**c**) Interaction information between pairs of *V*_FB_. Highly and lowly scored transitions appear as dark and bright pixels, respectively. In addition, an empty pixel represents no information in the database. Each value shown is linearly scaled to fall within the range of [0, 99].

We set the cost function, which evaluates the values of *V*_FB_, to map the *V*_FB_ schedule onto the Ising spin model. Here, the cost function is assumed to become large when the quantized conductance smoothly plateaus, and the step-wise conductance subsequently and clearly drops by *G*_0_ (*G*_0_ = 2*e*^2^/*h* = 77.6 μS, where *e* is the electron charge, and *h* is Planck’s constant). The time-dependent conductance curve corresponds to the change in the structure at the constricted section of the Au nanowires^33^. Therefore, we set five variables to represent the features of the conductance curve. Figure 2a shows a schematic of the conductance trace and applied voltage *V* and represents the variables in the cost function. *P*_1_ is the number of data points counted from *G*_ref_ in the quantized conductance that lie within a conductance window *G*_ref_ ± 0.5 *G*_0_ in each conductance trace, *L* is the number of data points from *G*_ref_ to the end of the FB cycle *G*_E_, *D* is the decrease in conductance in the region of EM occurrence, *F* is the decrease in conductance during one EM cycle between the start of the FB cycle *G*_S_ and *G*_E_, and *P*_2_ is the change in conductance between *G*_min_ and *G*_max_ during the application of ramp voltage. Furthermore, *N* is set as the number of voltage FB cycles during the FCE process. In an ideal control scheme, *D* and *F* are close to 1 *G*_0_, *P*_1_/*L*, which means the stability of the conductance trace, is set to 1, and *P*_2_ is set to 0 *G*_0_. Therefore, the cost function has the form

1 $$Scor{e}_{V{\rm{F}}{\rm{B}}}\equiv {s}_{n}(X)=\frac{{P}_{1}/L}{|D-1|+|F-1|+|{P}_{2}|}.$$

Here, we defined the output using Eq. (1) as the *Score* of *V*_FB_ values (*X*%) at *n*th times of FB. When the conductance trace is close to these ideal values, the *s*_*n*_(*X*) increases until infinity. Here, Eq. (1) is applied below 40 *G*_0_. In this region, the electron transport is in the ballistic regime and the EM is activated with the one-by-one removal of atoms, which is driven by the kinetic energy transfer from single conduction electrons to single Au atoms^34,35^. Hence, using Eq. (1), we can evaluate the *V*_FB_ values from the point of view of the conductance behavior under the voltage FB process.

Figure 2b presents the typical result of the conductance *G* of the Au nanowires and the voltage *V* as a function of the process time *t* when a random *V*_FB_ schedule is applied to an FCE. The vertical axis of Fig. 2b is the conductance *G* of the sample over *G*_0_. Furthermore, it should be noted that the decrease in the conductance of “1” *G*_0_ corresponds to a one-by-one removal of Au atoms^34,35^. Three FB points are indicated by black arrows, and Fig. 2b also presents the results of *s*_*n*_(*X*) obtained from Eq. (1). From Fig. 2b, the *s*_11_(40), in which *V*_FB_ = 40% at FB point 2, indicating smooth plateaus caused by quantized conductance, is much higher than *s*_10_(20) (at FB point 1) and *s*_12_(50) (at FB point 3). Therefore, the controllability of the quantized conductance can be evaluated with Eq. (1).

We quantify the correlation between the nearest-neighbor *V*_FB_ values through the following formula:

2 $$Scor{e}_{{\rm{t}}{\rm{r}}{\rm{a}}{\rm{n}}{\rm{s}}}\equiv {s}_{n,n+1}(X,Y)=\frac{{s}_{n}(X)+{s}_{n+1}(Y)}{2},$$

where the combination of *s*_*n*_(*X*) and *s*_*n*+1_(*Y*) represent *s*_*n*, *n*+1_(*X*, *Y*) occurring at *V*_FB_ of *X*% and *Y*% during an FCE process. In Fig. 2c, we show the mutual information between *V*_FB_ values. For example, *s*_1, 2_(10, 20) represents the evaluation for *V*_FB_ transition from 10% to 20% at the first to the second voltage FB cycles. In the FCE method, generally, Au nanowires should gradually narrow with the progress of FB cycles/steps, which is due to the EM. In other words, the conductance of Au nanowires should decrease with the evolution of FB processes. Here we hypothesize that a correlation exists between subsequent feedback steps because the EM proceeds by removing one atom at a time. Therefore, the correlation between nearest-neighbor *V*_FB_ values is assumed to be equivalent to considering the junction states between FB cycles. The pixels in Fig. 2c illustrate the fact that the transitions of the *V*_FB_ values strongly influence the evaluated values of an FCE process. As a result, we obtain the suitable combination of *V*_FB_ values for the controllability of quantized conductance during the FCE.

### Mapping of FCE schedules onto Ising spin model

We map the *V*_FB_ schedule onto the Ising spin model using the cost function described above. The formulation of *H*_Ising_, the system Hamiltonian (total energy of the system), for the Ising spin model can be described by

3 $${H}_{{\rm{Ising}}}=-\,\sum _{i}\sum _{x}\sum _{j}\sum _{y}{J}_{ix,jy}{\sigma }_{i,x}{\sigma }_{j,y}-\sum _{i}\sum _{x}{h}_{i,x}{\sigma }_{i,x}.$$

Typically, a real-world problem formulation requires conversion to the Ising Hamiltonian. In this research, the *V*_FB_ schedule is mapped into fully connected two-dimensional Ising spin cells consisting of *N* (row) × 9 (column) spins. As a result, the minimum value of the system energy and the corresponding optimum provide the solution to the original problem. In this model, each row corresponds to the order of *V*_FB_ values (*i*, *j* ∈ 1, …, *N*) and each column corresponds to the *V*_FB_ values (*x*, *y* ∈ 1,…, 9). Each spin (*σ*_*i*, *x*_ or *σ*_*j*, *y*_) takes a binary value of “+1” or “0,” which represents an up spin or a down spin. Furthermore, a mapped *V*_FB_ schedule is finally represented by a trace of spin value “+1.” The strength of the connection between spin (*i*, *x*) and spin (*j*, *y*) is denoted by *J*_*ix*, *jy*_. Each spin is also subject to the external magnetic field of *h*_*i*, *x*_. The formulation of the Ising Hamiltonian for the optimization of the FCE process is explained below. First, to select the highly rated schedules, the term

4 $${H}_{{\rm{evaluation}}}=\sum _{i}\sum _{x}\sum _{j}\sum _{y}{\delta }_{i,j-1}{W}_{x,y}{\sigma }_{i,x}{\sigma }_{j,y}$$

is set into the system Hamiltonian, where *W*_*x*, *y*_ is based on Eq. (2). For example, *W*_1, 2_ is decided based on the evaluation of the transition of *V*_FB_ from 10% to 20%. In addition, *δ*_*i*, *j*_ is equal to 1 (*i* = *j*) or 0 (otherwise). Penalty terms are added such that spin states that do not correspond to valid solutions are penalized. Then, to ensure no two *V*_FB_ overlap, we introduce a penalty term,

5 $${H}_{{\rm{overlap}}}=\sum _{i}\sum _{x}\sum _{j}\sum _{y}{\delta }_{i,j}{\sigma }_{i,x}{\sigma }_{j,y}.$$

Finally, to ensure that the total number of spins on “+1” is exactly *N*,

6 $${H}_{{\rm{total}}}=\sum _{i}\sum _{x}\sum _{j}\sum _{y}{\delta }_{i,j}{\delta }_{x,y}{\sigma }_{i,x}{\sigma }_{j,y}$$

is added to the system Hamiltonian. Hence, the cost function for *V*_FB_ scheduling, which is represented by *H*_FCE_, is given as

7 $${H}_{{\rm{FCE}}}=\frac{A}{2}{H}_{{\rm{evaluation}}}+\frac{B}{2}{H}_{{\rm{overlap}}}+\frac{C}{2}{H}_{{\rm{total}}}-D\sum _{i}\sum _{x}{h}_{i,x}{\sigma }_{i,x},$$

where *A*, *B*, *C*, and *D* are positive constants. A valid solution will have *H*_overlap_ = *H*_total_ = 0.

### Solution searches of FCE experimental parameters using Ising spin computing

Here, we first provide and set the optimal solution for our simulation to measure the performances of our Ising spin computing. According to the database, which consists of 50 samples, the average number of FB steps in the FCE process in a sample was approximately ten. Therefore, in the calculation experiments, we set 90 spins (*N* = 10, *N* × 9 = 90) with 10,000 iterative cycles. A single iterative cycle describes the flips of all spins chosen at random. Furthermore, the update of spins depends on the temperature *T* through the simulated annealing procedure^31^ with the aim of escaping from a local minimum. In each step of this algorithm, a spin is given a small random displacement and the resulting change, Δ*H*, in the energy of the system is computed. The probability that the configuration is accepted is *P*(Δ*H*) = exp(−Δ*H*/*T*). Random numbers uniformly distributed in the interval (0, l) are selected and compared to *P*(Δ*H*). If they are less than *P*(Δ*H*), the new configuration is retained; if not, the original configuration is used to start the iteration.

**Figure 3:**
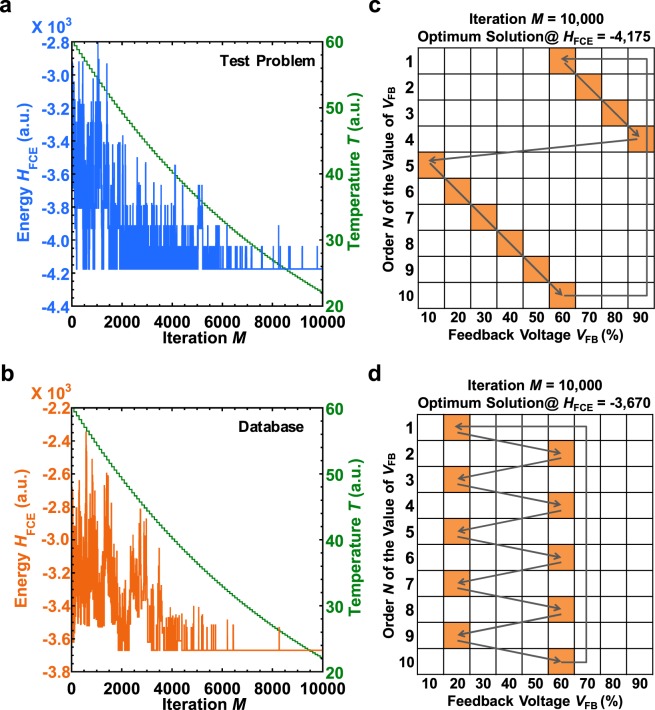
Optimization of FCE schedule using ground-state searches. Energy history of the ground-state search of the Ising spin model (**a**) for the optimal solution that we provide and specify the preset (blue), and (**b**) for the experimental data stored in the database (orange). The annealing schedule (green) decreases nonlinearly. Our simulated annealing algorithm uses single spin-flip Metropolis updates with a linear profile of inverse temperatures *β* = *T*^−1^, where *β* is calculated by 60 × 0.99 (with *β* updated every 100 iterative cycles over the lattice spins). The energy profiles of (**a**,**b**) exhibit similar characteristics (*A* = 5, *B* = 430, *C* = 550, *D* = 360, *h*_i, x_ = 1). Final spin status after 10,000 iterative cycles for the (**c**) test problem and (**d**) database. Spin states in orange and white represent up spin and down spin states, respectively. Here, the optimal solution of our simulation is identified as follows: *V*_FB_(%) = 60 → 70 → 80 → 90 → 10 → 20 → 30 → 40 → 50 → 60 in the (**c**) (test problem) and *V*_FB_(%) = 20 → 60 → 20 → 60 → 20 → 60 → 20 → 60 → 20 → 60 in the (**d**) (database).

In the test problem we set, at the global minimum with a system energy *H*_FCE_ of −4,175, the final spin status appears and traces the following: *V*_FB_(%) = 60 → 70 → 80 → 90 → 10 → 20 → 30 → 40 → 50 → 60. Figure 3a shows the system energy *H*_FCE_ and temperature *T* as a function of the number of iterative cycles. In this case, the computation is complete within seconds because we used conventional computing resources. Over time the energy decreases and is characterized by upward and downward movements. After 10,000 iterative cycles, the system energy reaches −4,175. Therefore, the system energy is considered to have reached the global minimum we set through the ground-state searches of the Ising spin computing. Figure 3c also presents the final spin status of the system at the global minimum energy. Each spin is expressed by a square. The spin state in orange in Fig. 3c represents the optimal solution set of *V*_FB_ scheduling with *H*_FCE_ = −4,175. Based on these results, Ising spin computing can determine the optimal solution of experimental parameters for the *V*_FB_ schedule.

To demonstrate the utility of our Ising spin computing, we apply it to a database containing conductance measurements of 50 Au nanowires. Figure 3b plots the process of the ground-state search based on the experimental data stored in the database. As seen in Fig. 3b, the energy profiles exhibit similar characteristics to those in Fig. 3a. Furthermore, the final ground-state spin configuration in Fig. 3d represents a suitable *V*_FB_ schedule that alternates between *V*_FB_ of 20% and 60% with time. This result traces the spins with highly evaluated values of *V*_FB_ obtained from the database consisting of 50 samples. Thus, Ising spin computing progressed from the entirely random data of the *V*_FB_ schedule towards an indication of the experimental parameters for the *V*_FB_ schedule. Clearly, an ordered *V*_FB_ schedule can be generated from the disordered data stored in the database by using the proposed Ising spin computing model.

### Score of *V*_FB_ schedules obtained with solution searches

Next, we investigated the ability of our Ising spin computing to search for the ground-state solution. We did this by comparing the score of the *V*_FB_ scheduling obtained from the Ising spin computing and random selection. Here, we defined the *Score* of *V*_FB_ schedule as

8 $$Scor{e}_{{\rm{p}}{\rm{r}}{\rm{o}}{\rm{c}}{\rm{e}}{\rm{s}}{\rm{s}}}\equiv {S}_{N}=\mathop{\sum }\limits_{n=1}^{N}{s}_{n,n+1}(X,Y).$$

**Figure 4:**
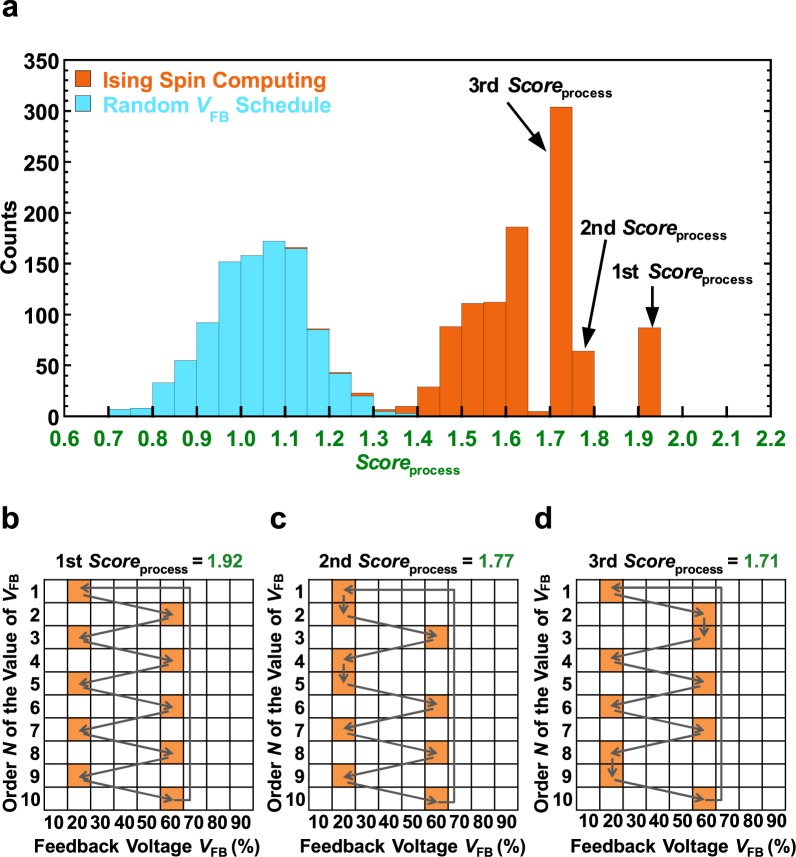
Ability of Ising spin computing to search for the ground-state solution. (**a**) Number of Ising spin computing (orange) and random (blue) cases versus score of *V*_FB_ schedule (*Score*_process_) calculated from database (*A* = 5, *B* = 430, *C* = 550, *D* = 360, *h*_i, x_ = 1, *β* = 60). The values of *Score*_process_ for Ising spin computing and random cases are obtained by adding 10 times of *Score*_trans_ for each *V*_FB_ schedule obtained by each method. (**b**–**d**) The top three final spin statuses corresponding to each score out of (**a**). All statuses are the results obtained as output from Ising spin computing, and the highest rated (**b**) is the optimal *V*_FB_ schedule produced from the database.

Notice that *s*_n,n+1_ (*X*, *Y*) is *s*_*N*,1_ (*X*, *Y*) when *n* = *N*. In other words, Eq. (8) evaluates the entire *V*_FB_ scheduling. Because *N* is set to 10 in this study, a *V*_FB_ schedule consisting of 10 FBs is evaluated by Eq. (8), and *S*_10_ is calculated. The detailed calculation procedure of *S*_10_ is as follows. First, a *V*_FB_ schedule consisting of 10 FBs is generated with Ising spin computing or random selection. Next, *s*_1,2_, *s*_2,3_, …, and *s*_10,1_ are scored by the database shown in Fig. 2c. Finally, *S*_10_ is calculated by adding *s*_1,2_, *s*_2,3_, …, and *s*_10, 1_. Figure 4a presents a histogram of the *Score*_process_ obtained from the Ising spin computing (orange histogram) and random selection (blue histogram) when both are performed 1,000 times. All initial spin values were random in all experiments described in this paper to explore the various conditions. The blue histogram in Fig. 4a exhibits uniform Gaussian-like score distributions, which represent the validity of the database. In addition, the distribution of the histogram shifting to the right indicates that the highly rated *V*_FB_ schedule is selected. From Fig. 4a, the orange histogram of the Ising spin computing is significantly shifted to the right as compared with the histogram obtained from random selection. This result indicates that Ising spin computing can automatically select the highly evaluated *V*_FB_ schedules through the ground-state searches of the solutions. Figure 4b–d show the top three final spin statuses corresponding to each *Score*_process_. The highest scoring solution (Fig. 4b) is considered the optimum schedule of *V*_FB_. Further, the results of the scoring solutions for the second and third place in Fig. 4c,d indicate that the heuristic solutions almost agree with the optimal schedule. Therefore, the local minimum solutions obtained by Ising spin computing are also considered suitable for use in the FCE method. These results show that Ising spin computing could discover a remarkable amount of information about the FCE by starting from disorderly generated data; that is, these are the results of applying machine learning to disordered data to obtain order in exploring the experimental parameters of the FCE method.

### FCE experiments with results of ground-state searches of experimental parameters

**Figure 5:**
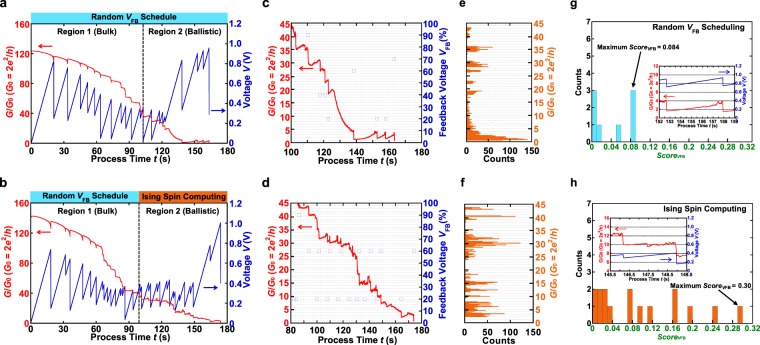
Comparison of Ising spin computing method with random method. (**a**,**b**) Time evolution of conductance for Au nanowires during the FCE process. A random *V*_FB_ schedule is applied for both methods in the range of *G* ≥ 40 *G*_0_ (region 1). In the range of *G* ≤ 40 *G*_0_ (region 2), the random *V*_FB_ schedule is applied in (**a**) and the optimal *V*_FB_ schedule obtained by the ground-state searches of the Ising spin model is applied in (**b**). (**c**,**d**) Conductance traces recorded for ballistic regime (*G* ≤ 45 *G*_0_). (**e**,**f**) Conductance histograms obtained for (**c**,**d**), respectively. Because of the constant plateaus, the conductance histogram in (**f**) exhibits sharp peaks at integer multiples of the conductance quantum compared with (**e**). (**g**,**h**) Distributions of the *Score*_*V*FB_ constructed from the region 2 in (**a**,**b**), respectively. The insets in (**g**,**h**) are conductance curves showing the largest *Score*_*V*FB_.

Figure 5a,b present the conductance traces recorded during the FCE with the schedule of the 1st *Score*_process_ obtained from the Ising spin computing and random *V*_FB_ scheduling, respectively, which are prepared as a reference. In the initial stage denoted as region 1, where the conductance is larger than 40 *G*_0_, the conductance decreases continuously because of the EM of Au atoms. This result indicates that region 1 shows the bulk regime of electrical conduction. However, as the conductance decreases to less than 40 *G*_0_, conductance plateaus and steps at and near integer multiples of 1 *G*_0_ are observed, as seen in region 2. This result implies that region 2 shows the ballistic conduction regime and that EM proceeds with the behavior of the one-by-one removal of single Au atoms^34,35^. Here, to compare both methods from the viewpoint of the controllability of conductance quantization, enlarged views of region 2 are shown in Fig. 5c,d. The conductance changes in steps, consisting of plateaus and jumps. Further, Fig. 5e,f present histograms of the conductance values derived from the EM data of Fig. 5c,d, respectively, in the range *G* ≤ 45 *G*_0_. As the conductance changes whenever the atomic structure changes at the metal contacts, stable structures can yield preferred peaks in the conductance histograms^33^. Therefore, the sharp peaks indicate that particular conductance values and their corresponding contact configurations are more stable. Conductance plateaus and steps at and near integer multiples of *G*_0_ are observed in Fig. 5f as compared to Fig. 5e. We further applied Eq. (1) to each conductance trace in the range *G* ≤ 40 *G*_0_ to compare *V*_FB_ scheduling in terms of *Score*_*V*FB_. Figure 5g,h show the *Score*_*V*FB_ and the insets show the highest evaluated conductance traces. As shown in Fig. 5g,h, the results of the optimal *V*_FB_ scheduling obtained from Ising spin computing are superior to those from random *V*_FB_ scheduling not only from the conductance histograms but also from the viewpoint of *Score*_*V*FB_. In addition, the conductance trace in the inset of Fig. 5h shows conductance plateaus around 10 *G*_0_. These results suggest that the ballistic conduction regime in the electrical conduction is precisely controlled by the *V*_FB_ scheduling obtained by the ground-state solution of the Ising spin computing, despite the complicated phenomenon of mass transport in electromigrated nanowires.

**Figure 6:**
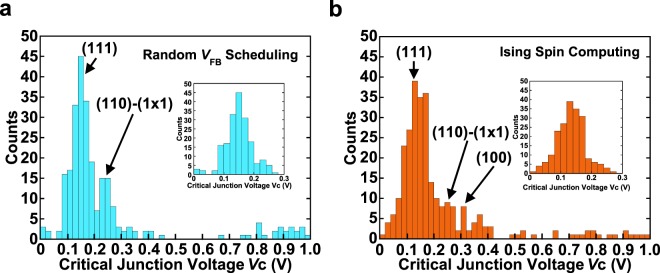
Histogram of the critical values of the junction voltage, *V*_c_. The histograms are constructed from the results of applying a (**a**) completely random *V*_FB_ schedule and (**b**) *V*_FB_ schedule, where the optimal parameters are applied at *G* ≤ 40 *G*_0_. The insets in (**a**,**b**) are histograms of the *V*_C_ accumulated for the region of *G* ≥ 40 *G*_0_. The major peaks agree well with the surface self-diffusion potentials of Au atoms. A remarkable finding is that the large peak at 0.12–0.14 eV coincides with the activation energy for self-diffusion on the (111) surfaces^36^. The small peak visible at 0.26–0.28/0.24–0.26 eV is close to the self-diffusion on the (110)-(1 × 1) surfaces predicted by molecular dynamics simulations^37^. Furthermore, 0.3–0.32 eV is close to the theoretically predicted activation energy for the self-diffusion energy on the (100) surfaces^38^.

To gain insights into the dynamics of EM in Au nanowires, we accumulated *V*_C_ and plotted its histogram in Fig. 6a (applied using random *V*_FB_ schedule) and Fig. 6b (applied using optimized *V*_FB_ schedule). *V*_C_ is the maximum value of the junction voltage *V*_J_ for each FB cycle^34,35^. Each histogram consists of data collected from 22 samples. In addition, each inset in Fig. 6a,b shows a histogram constructed from the *V*_C_ accumulated for the region of *G* ≥ 40 *G*_0_. Umeno *et al*.^34,35^ reported that *eV*_C_ can be regarded as “the chemical potential for atom migration” and, hence, the obtained histogram can be interpreted as “the spectrum of electromigration”. As seen in Fig. 6a,b, major peaks coincide with the activation energies for the surface potential of Au adatoms^36–38^, results that are similar to those in ref.^35^. Hence, Fig. 6a,b clearly indicate that the process of EM at the Au nanowires is driven by the microscopic kinetic energy transfer from a single conduction electron to a single metal atom^34,35^. In addition, the difference between Fig. 6a,b appears in the range from 0.26 to 0.4 eV, showing a major peak at 0.3–0.32 eV in the case of Fig. 6b. This value is equal to the activation energy for the self-diffusion on the (100) surfaces, which was predicted by the embedded atom model^38^. This is due to the difference in the *V*_FB_ scheduling in the ballistic regime (*G* ≤ 40 *G*_0_) between the random *V*_FB_ schedule (Fig. 6a) and the *V*_FB_ schedule optimized by Ising spin computing (Fig. 6b). The findings obtained here provide a solid basis for understanding the migration of Au atoms at atomic junctions driven by a controlled EM method.

## Conclusions

In this study, we established a novel design method for determining FCE parameters using Ising spin computing. Optimization of the parameters of the FCE process using the Ising spin computing method provides an alternative to the conventional method with human intervention that requires expert knowledge of the physical processes and methods involved. The advantages of using the Ising spin model over traditional machine-learning methods for small training datasets have been reported^27,28^. Further, we showed that the FCE process can be optimized with a small database of only 50 samples. Therefore, in areas of research where datasets with a small number of relevant samples may be more common, Ising spin computing may be the algorithm of choice. However, this improvement could be even greater when expanding the database and/or optimizing more FCE parameters such as *G*_TH_ and *V*_STEP_, using Ising spin computing because the database is too small. In fact, as the size of the training dataset increased, the results improved to a certain degree in “a Higgs optimization problem^27^” and “a simplified computational biology problem^28^.” Furthermore, in the case of maximum-cut problems, the accuracy of the solutions remained almost constant for various problem sizes^39^. Hence, the same tendency is expected to be obtained in FCE parameter optimization with Ising spin computing.

In this study, ground-state searches of Ising spin computing were performed by simulated annealing, which is a classical method. However, in the future, the development of quantum annealers may make it possible to optimize the experimental parameters of FCE using quantum annealing considering that various optimization problems have been solved with commercially available quantum annealers^23–30^. Therefore, the quantum annealing technique is expected to provide appropriate solutions or optimize the experimental parameters of FCE. Furthermore, the Ising spin model features intrinsically nonlocal correlations that can lead to substantially more compact representations of many-body quantum states. Therefore, their effectiveness for the optimization of the experimental parameters of the FCE method makes them a powerful tool for applications beyond the formation of nanogaps and/or controlling quantized conductance in nanowires, including understanding complicated quantum-mechanical systems.

## Methods

### Au nanowire fabrication and design

A Si wafer with a thermally grown SiO_2_ layer is used as the substrate; next, a positive tone resist was spin coated onto the substrate. The electrode pattern with a constriction of ~100 nm was fabricated using electron-beam lithography. A standard development procedure was applied to remove the resist by inserting the substrate into a development solution, after which the substrate was transferred into propanol to terminate the development process. The resist layer served as a mask for deposition of the metal layer, after which the sample was immersed in acetone for the lift-off process to remove the redundant part of the metal layer.

### Electrical characterization

A source-measure unit was used for sourcing the voltage (up to 10 V and with 50 μV sensitivity) and measuring the voltage (up to 10 V with 10 μV sensitivity) and the current (up to 10 mA with 10 nA sensitivity), allowing the characterization of current–voltage behavior with a time resolution of 25 ms. All measurements were carried out under a two-terminal arrangement. The FCE experiments for the Au nanowires were performed at room temperature under ambient conditions using a real-time operating system (RTOS)-based controller, which is specifically designed to run FCE applications with precise timing and reliability. The system consisted of a host PC, RTOS, and target samples of Au nanowires. In this system, we first designed and coded the FCE algorithm on a host PC before deploying the algorithm in the RTOS. As a result, the embedded algorithm with a user-defined program could be performed with precise timing and reliability.

### Ising spin computing

*W*_*x*,*y*_ is calculated by

9 $${W}_{x,y}=100-\frac{{\rm{a}}{\rm{v}}{\rm{g}}\,Scor{e}_{{\rm{t}}{\rm{r}}{\rm{a}}{\rm{n}}{\rm{s}}}(X,Y)-\,min\,Scor{e}_{{\rm{t}}{\rm{r}}{\rm{a}}{\rm{n}}{\rm{s}}}(X,Y)}{max\,Scor{e}_{{\rm{t}}{\rm{r}}{\rm{a}}{\rm{n}}{\rm{s}}}(X,Y)-\,min\,Scor{e}_{{\rm{t}}{\rm{r}}{\rm{a}}{\rm{n}}{\rm{s}}}(X,Y)}\times 99,$$

where max*Score*_trans_ (*X*, *Y*) and min*Score*_trans_ (*X*, *Y*) are maximum and minimum values of avg*Score*_trans_ (*X*, *Y*) in the database, respectively, and avg*Score*_trans_ (*X*, *Y*) represents the average of each *Score*_trans_ (*X*, *Y*). By using Eq. (9), when avg *Score*_trans_ (*X*, *Y*) is the largest and the smallest, *W*_*x*, *y*_ becomes 1 and 100, respectively. *W*_*x*, *y*_ is set to 100 in case of no information of *Score*_trans_ (*X*, *Y*). In our formulation of the Hamiltonian, each term (*A*, *B*, *C*, and *D*) is derived heuristically, based on the size of the problem. At the beginning, the values of *C* and *D* are set to an initial value such that the number of up spins would be *N* (with the values of *A* and *B* set to 0). Then, each row has exactly one up spin by setting the value of *B*. Finally, the values of *A* are gradually increased within the range where valid solutions are obtained while adjusting the value of *D*. In the test described in the text, ground-state searches were carried out 100 times with the optimal solution set to *H*_FCE_ of −4,175 to check the performance of our Ising spin computing (*W*_*x*,*y*_ is set to 1 or 50). As a result, the global minimum (*H*_FCE_ = −4,175) and the optimum solution are obtained at a rate of 95%.

### Database and experimental results of FCE

The number of data obtained from 50 samples was 511 FBs (*G* < 100 *G*_0_), and the maximum FB number obtained in one sample was 22. The ratio of *V*_FB_ = 10, 20, 30, 40, 50, 60, 70, 80, and 90% in the database is 14.3, 10.8, 9.98, 11.2, 9.59, 11.7, 13.7, 9.00, and 11.4%, respectively. The design values for length and width of nanowires are 650 and 85 nm, 500 and 50 nm, and 500 and 100 nm. The data in Fig. 5 were obtained from Au nanowires with the length and width of approximately 300 and 100 nm. In our case, the initial resistance *R* of samples was typically amounted to around 74–190 Ω. This was measured by sweeping the voltage from –7 to 7 mV in steps of 100 μV. The average value of *Score*_*V*FB_ in the database, Fig. 5g,h is 0.042, 0.040, and 0.096, respectively.
